# Outcomes After Surgical Treatment of Infective Endocarditis with Destruction of the Cardiac Skeleton

**DOI:** 10.3390/medicina62010033

**Published:** 2025-12-24

**Authors:** Mascha von Zeppelin, Andreas Winter, Fabian Emrich, Zdenka Holubcova, Florian Hecker, Jan Hlavicka, Hiwad Rashid, Thomas Walther, Tomas Holubec

**Affiliations:** Department of Cardiovascular Surgery, University Hospital, Goethe University Frankfurt, 60590 Frankfurt am Main, Germany; mascha.vonzeppelin@unimedizin-ffm.de (M.v.Z.); andreas.winter@unimedizin-ffm.de (A.W.); fabian.emrich@unimedizin-ffm.de (F.E.); zdenka.holubcova@unimedizin-ffm.de (Z.H.); florianhecker@gmx.de (F.H.); jan.hlavicka@unimedizin-ffm.de (J.H.); hiwad.rashid@unimedizin-ffm.de (H.R.); thomas.walther@herz-frankfurt.de (T.W.)

**Keywords:** infective endocarditis, surgical treatment, commando and hemi-commando procedure, long-term outcomes

## Abstract

*Background and Objectives*: Infective endocarditis (IE) continues to represent a life-threatening clinical entity, particularly in patients with advanced involvement of the cardiac fibrous skeleton. This study was designed to determine the incidence and to evaluate both short- and long-term outcomes in patients undergoing complex surgical intervention necessitating patch reconstruction for extensive and destructive IE. *Materials and Methods*: Between January 2008 and December 2024, 678 patients underwent cardiac surgery for IE at University Hospital Frankfurt/Main. The primary endpoint was long-term survival; the secondary endpoint was freedom from reoperation. *Results*: Ninety-six patients (14%) required complex patch reconstruction, owing to the severe involvement of the cardiac fibrous skeleton. The median age was 68 years (interquartile range [IQR], 16.5 years). Forty-three patients underwent redo surgery following previous cardiac procedures. Abscess formation was identified in 88% of cases (n = 85). Infective endocarditis was predominantly left-sided in 97% of patients (n = 94). In 40 patients (41%), the aortomitral continuity or the left ventricular outflow tract (LVOT) was involved. Combined surgical procedures were performed in 85 patients (87.6%), including 19 commando or hemi-commando operations. Thirty-day mortality was 20% (n = 19). The estimated 5- and 10-year survival rates were 46.5 ± 5.5% and 26.1 ± 6.8%, respectively. Survival did not differ significantly between native and prosthetic valve endocarditis, nor between commando/hemi-commando procedures and cases with abscess formation but preserved aorto-mitral continuity. *Conclusions*: In industrialized countries, extensive IE with abscess formation or destruction of the cardiac skeleton is predominantly associated with *Staphylococcus aureus*. Patients undergoing commando or hemi-commando procedures do not experience inferior survival compared with other patients with extensive IE. No survival advantage was observed for native versus prosthetic valve IE in the presence of extensive abscess formation.

## 1. Introduction

The incidence of infective endocarditis (IE) has remained largely stable in recent years; nevertheless, diagnosis and treatment continue to pose major challenges. Increasing patient age and advances in medical care have led to a higher prevalence of risk factors, including valvular heart disease with valve replacement, intracardiac devices, diabetes mellitus and hemodialysis, etc. [1,2,3]. Despite ongoing progress in medical therapy, IE remains associated with substantial morbidity and mortality [4,5]. More than 40% of patients with infective endocarditis require surgical intervention, which remains associated with perioperative mortality rates of up to 30%. Accurate pathogen identification and antibiotic susceptibility testing are essential for appropriate management, extending beyond the surgical setting [6].

The cardiac fibrous skeleton is a connective-tissue framework located at the level of the heart valves. On the one hand, it separates the atria from the ventricles, and on the other hand, it attaches the heart valves, separating the atria from the ventricles and providing structural support for valvular attachment. It consists of the fibrous annuli, fibrous trigones and the membranous septum. A major surgical challenge in IE is abscess formation, which, in severe cases, leads to the destruction of the intervalvular fibrosa (IVF). Abscesses in this region disrupt the continuity between the mitral valve and the aortic root, rendering isolated valve replacement insufficient. Radical debridement, removal of prosthetic material and drainage of the abscess cavities are mandatory [7]. Although infection control can be achieved, the resulting tissue destruction frequently necessitates complex reconstructive procedures, as the secure anchoring of prosthetic valves is no longer possible. Consequently, combined aortic and mitral valve replacement with patch reconstruction is often required, and these procedures are associated with substantially higher morbidity and mortality than conventional cardiac surgery [8,9]. Because such operations are rare, published data remain limited.

The aim of this study was to evaluate early and long-term outcomes after surgical treatment of extensive IE complicated by abscess formation and partial destruction of the cardiac fibrous skeleton.

## 2. Materials and Methods

### 2.1. Study Design

**Figure 1:**
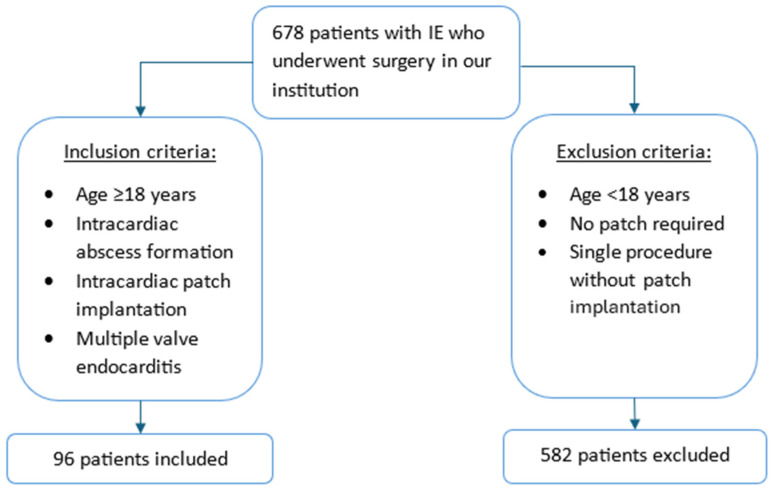
Study design with inclusion and exclusion criteria (IE—infective endocarditis).

Between January 2008 and December 2024, a total of 678 patients undergoing surgery for acute or subacute IE were identified from a prospective institutional database. The diagnosis of IE was established according to the Duke criteria [10]. Among these patients, 96 exhibited intracardiac abscess formation with partial destruction of the cardiac skeleton, requiring intracardiac patch reconstruction (Figure 1). Destruction involved the intervalvular fibrosa (IVF) and/or the left ventricular outflow tract (LVOT). The remaining 582 patients were excluded because patch reconstruction was not required or only a single valve was involved. This study was approved by the Institutional Ethical Committee (reference number: 2024–2048; approval date: 11 December 2024). The requirement for informed consent was waived due to the retrospective design.

### 2.2. Surgical Procedure

Surgical access was obtained either through a conventional median sternotomy or a partial upper sternotomy (J-shaped incision extending into the third or fourth intercostal space). A cardiopulmonary bypass (CPB) with standard cardioplegic cardiac arrest was established in all patients.

Arterial cannulation for CPB was performed via the ascending aorta, femoral artery or axillary artery, as dictated by anatomical and clinical considerations. Venous cannulation was achieved through the right atrium, bicaval cannulation or the femoral vein. Following the initiation of CPB, the aorta was cross-clamped and blood cardioplegia was administered. In patients with destruction of the intervalvular fibrosa, reconstruction was performed using a pericardial patch, combined either with double-valve replacement (commando procedure) or aortic valve (AV) replacement with mitral valve (MV) repair (hemi-commando procedure). First, the aorta is incised, directed towards the middle of the non-coronary sinus, and an additional incision is made in the left atrial roof, extending from the level of the right pulmonary artery to the base of the anterior mitral leaflet (AML) [11].

**Figure 2:**
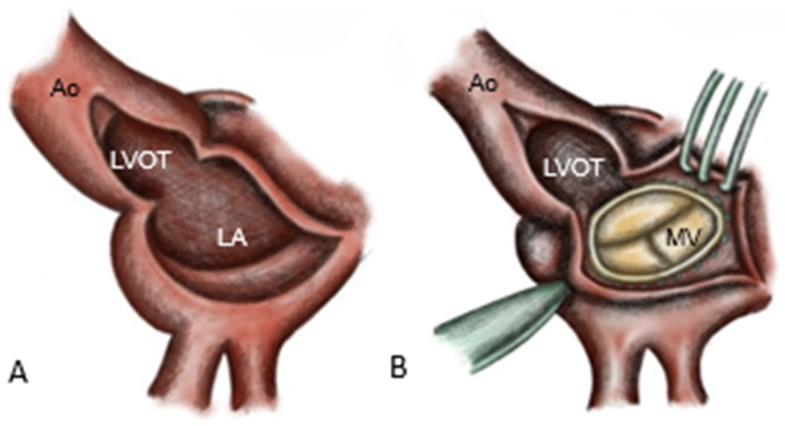
Surgical technique for commando procedure; (**A**) view of the left atrium and the left ventricular outflow tract; (**B**) replaced mitral valve with bioprosthesis. Ao: aorta; LVOT: left ventricular outflow tract; LA: left atrium; MV: mitral valve replacement (bioprosthesis).

The infected AV, the aortic root, the left atrial roof and the base of the anterior MV leaflet are excised [8], resulting in complete resection of the intervalvular fibrosa. Resection is extended to healthy tissue with a safety margin of approximately 3–5 mm, simultaneously allowing the drainage of the abscess cavities into the left ventricle. The mitral valve prosthesis is implanted first in the posterior annulus (Figure 2).

**Figure 3:**
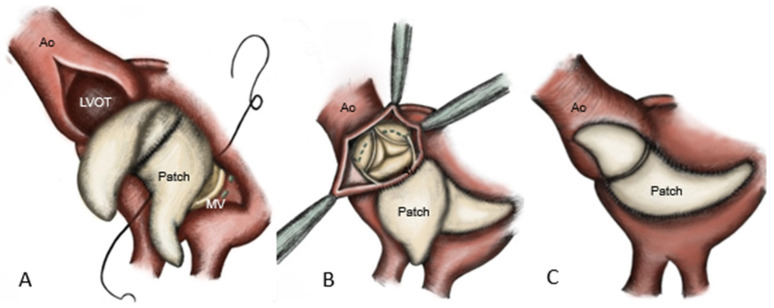
Surgical technique of commando procedure: (**A**) reconstruction of the intervalvular fibrosa with bovine pericardial patch. The anterior part is sutured into the mitral valve: (**B**) reconstruction of the LA roof, after aortic valve replacement was carried out and (**C**) reconstruction of the aortic root. Ao: aorta; LVOT: left ventricular outflow tract; MV: mitral valve replacement (bioprosthesis).

Reconstruction of the aorto-mitral curtain was achieved by using a folded glutaraldehyde-fixed bovine pericardial patch, which was sutured anteriorly to the mitral prosthesis [8] and posteriorly to the atrial roof. The remaining patch is anchored to the LVOT and ascending aorta [12], followed by the implantation of the aortic prosthesis and closure of the aortotomy (Figure 3).

Additional procedures, including aortic root replacement, are performed as indicated. In cases without IVF involvement, abscesses were drained and covered with a pericardial patch. If several heart valves were affected, they were reconstructed or replaced, depending on the extent of the damage. Following successful surgical reconstruction, myocardial reperfusion was initiated with the administration of a terminal warm blood cardioplegia (“hot shot”) for 3 min, followed by the release of the aortic cross-clamp. After intraoperative assessment by transesophageal echocardiography, patients were weaned from the cardiopulmonary bypass and the chest was subsequently closed in a standard fashion. Transesophageal echocardiography was routinely employed to evaluate prosthetic and native valve competence.

### 2.3. Microbiological Methods

Following intraoperative collection, valve tissue specimens were divided into two aliquots: one designated for a conventional microbiological culture and the other for polymerase chain reaction (PCR) analysis. Prior to the culture preparation, tissue specimens were homogenized. Cultures were performed on solid media, including aerobic blood agar, anaerobic blood agar, chocolate agar (5% CO_2_), MacConkey agar and Sabouraud agar, using a three-phase streaking technique. In addition, tissue homogenates were inoculated into liquid enrichment media, including brain–heart infusion broth and thioglycolate broth. Incubation was conducted at 36 ± 1 °C for a total duration of 14 days [13].

For PCR analysis, tissue specimens were digested with tissue lysis buffer and proteinase K, followed by DNA extraction using a commercially available kit. Bacterial identification via PCR was performed by the amplification and sequencing of the 16S rRNA gene, employing primers targeting either the 3′ end (16S-F: 5′-CAAACAGGATTAGAGATACCC) or the 5′ end (16S-R: 5′-CCCGGGAACGTATTCACCG) of the 16S rDNA, as previously described. Real-time PCR was conducted under the following cycling conditions: initial denaturation at 95 °C for 2 min, followed by 40 cycles of denaturation at 95 °C for 5 s, annealing at 55 °C for 20 s and extension at 72 °C for 30 s [13].

### 2.4. Data Collection and Follow-Up

Demographic, intraoperative and postoperative data were collected retrospectively and entered into a database. Follow-up was conducted via structured telephone interviews with patients and their general practitioner, or by mailed questionnaires. Follow-up ended in December 2024.

### 2.5. Statistical Analysis

The data were entered into a database using Microsoft Office Excel^®^ (Version 2010 for Windows; Microsoft Corp, Redmond, WA, USA). The Kolmogorov–Smirnov test was used to assess the normality distribution of the data. Continuous variables are presented as mean ± standard deviation or a median with range for non-normally distributed data. Categorical variables are reported as frequencies and percentages. The Pearson chi-square test was used to compare categorical variables. Survival time was estimated using the Kaplan–Meier survival methods. An univariable and multivariable Cox proportional hazards regression was performed to assess the association with mortality to elaborate on the factors associated with increased long-term mortality. The results of the Cox model were expressed as a hazard ratio (HR) with a 95% confidence interval (CI).

The statistical analysis was performed with Microsoft Office Excel^®^ (version 2010 for Windows; Microsoft Corp, Redmond, WA, USA) and SPSS software (IBM Corp. Released 2017. IBM SPSS Statistics for Macintosh, version 25.0. Armonk, NY, USA: IBM Corp.), where a *p*-value below 0.05 was considered to be statistically significant.

Missing values were analyzed using multiple imputation and proportional hazard assumptions were checked with the Cox model.

## 3. Results

### 3.1. Study Population and Clinical Data

Median patient age was 68 years (IQR 16.5); 30% (n = 29) were female and 70% (n = 67) were male (Table 1).

Vegetations were detected preoperatively in 91 patients (94%) via echocardiography. In 35% (n = 34), preoperative CT confirmed that cerebral infarcts were present, which were related to the IE. Surgery was performed emergently in 24% (n = 23), urgently in 66% (n = 63) and electively in 10% (n = 10) of patients. These were patients who were scheduled for surgical treatment due to a surgical valve problem, but endocarditis only became apparent intraoperatively.

The median time for diagnosis based on the Duke criteria was 4 days (IQR 7) and the median time in hospital until surgical treatment was 13 days (IQR 14.25). The median time from visualization on echocardiography to surgical treatment was 8 days (IQR 11).

Sixty-eight patients initially presented to a peripheral center; 28 patients presented directly to our center. The median time to echocardiographic diagnosis was shorter in peripheral centers (3 days, IQR 6) compared to patients admitted in domo (6 days, IQR 15). In contrast, the days from echocardiographic diagnosis to surgical treatment were comparable: peripheral, 7 days (IQR 14) and in domo, 8 days (IQR 8). A focus was identified in 74% (n = 71). In addition to prosthetic endocarditis, which certainly offers an increased risk of endocarditis, gastrointestinal foci (interventional procedures, chronic intestinal diseases) were almost on par with odontogenic foci (8.4%), urogenital (8.4%) and pulmonary (8.4%) (including COVID 19-associated infections) at 10% (n = 7). The following focus was subsequently identified: postinterventional procedures (occluder, stent, etc.), intravenous drug abuse, neurological abscess formations, phlegmon of the lower extremity (associated with peripheral arterial occlusive disease), surgical procedures (especially trauma surgery), infected implants and wound infections after cardiac surgery.

Blood cultures identified pathogens preoperatively in 62% (n = 60) of patients, with *Staphylococcus aureus* being the most common organism at 38% (n = 23), followed by *Enterococcus faecalis* with 15% (n = 9). Furthermore, *Staphylococcus epidermidis* (8%; n = 5) and *Streptococcus agalactiae* (5%; n = 3) were detected in >5% of cases. Only 76% (n = 74) of all patients received preoperative antibiotic therapy.

Eighty-one percent of patients had an indication for surgical treatment for AV, of which 77% had AV disease: 55% had AV insufficiency, 17% had AV stenosis and 5% had combined AV disease. Fifty-three percent were indicated for surgical MV replacement, of which 80% had valvular disease: 65% had insufficiency, 8% had stenosis and 7% had combined disease. Prosthetic valve endocarditis (PVE) was present in 45% (n = 43), and abscess formation was present in 88% (n = 85) of patients.

### 3.2. Intraoperative Data

The intraoperative data are presented in Table 2.

**Figure 4:**
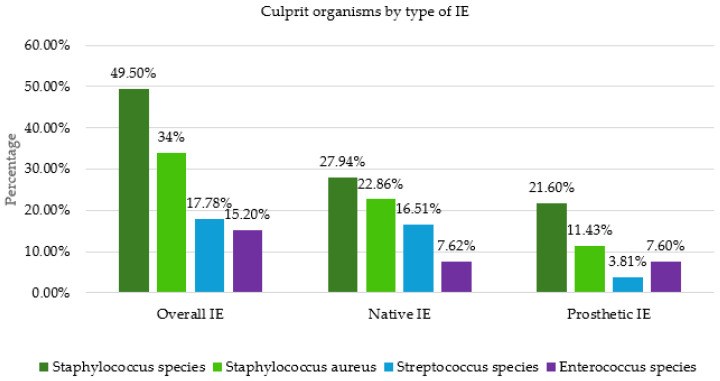
Culprit organisms by type of infective endocarditis. IE: Infective endocarditis.

Samples were obtained intraoperatively for further diagnostics in PCR analysis, culture and pathologic evaluation. In 79 patients (81%), it was possible to detect microorganisms from the samples obtained intraoperatively. In 48 patients (49%), the pathogen detection from the preoperatively collected blood cultures was consistent with the detection from the intraoperatively collected samples. Seventeen patients remained completely without pathogen detection. Staphylococci were the most common bacteria found in intraoperative samples (49%), with Staphylococcus aureus accounting for 34% of these (Figure 4). They were followed by streptococci and enterococci. We observed the same pattern in native valve endocarditis (NVE). In prosthetic valve endocarditis (PVE), staphylococci were also predominant, followed by enterococci.

### 3.3. Postoperative Outcomes

Re-exploration for bleeding was required in 26% (n = 25) of patients. Thirteen patients (13%) suffered from perioperative stroke, 3% from postoperative myocardial infarction. Low-cardiac-output syndrome was diagnosed in 30 patients (31%) and 27 patients required postoperative extracorporeal life support (ECLS). Multi-organ failure affected 21 patients (22%) postoperatively. The median ICU time was 4 days (IQR 7). Nineteen patients (20%) died in the first 30 days after operation.

### 3.4. Follow-Up

In the multivariable risk analysis, only preoperative renal insufficiency (HR 0.43 (95% CI 0.86); *p* = 0.01), intraoperative LVOT reconstruction (HR 2.8 (95% CI 9.18); *p* = 0.017) and postoperative multiple organ failure (HR4.6 (95% CI 17.50); *p* = 0.021) were found to be negative predictors for the endpoint of death. However, it should be noted that the evaluation can only be assessed to a limited extent, due to the small overall cohort. For this reason, we have also created a single regression model, even though the data are less meaningful than in a multivariate risk analysis (Table 3). In this model, preoperative renal insufficiency was the only factor associated with higher mortality (HR0.50 (95% CI 0.91); *p* = 0.025).

**Figure 5:**
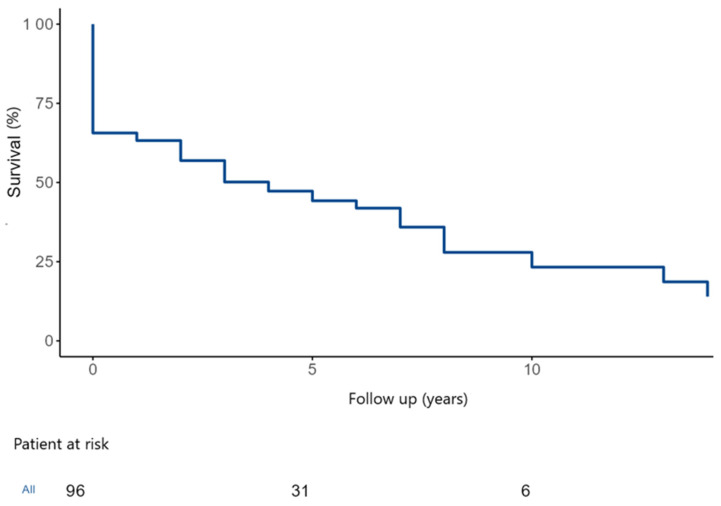
Kaplan–Meier curve showing the overall survival after surgical treatment for extensive IE with abscess formation and partial destruction of the heart skeleton. IE: Infective endocarditis.

During the study follow-up, 55 patients (57%) died. The overall estimated 5- and 10-year survival were 46.5 ± 5.5% and 26.1 ± 6.8% (Figure 5).

**Figure 6:**
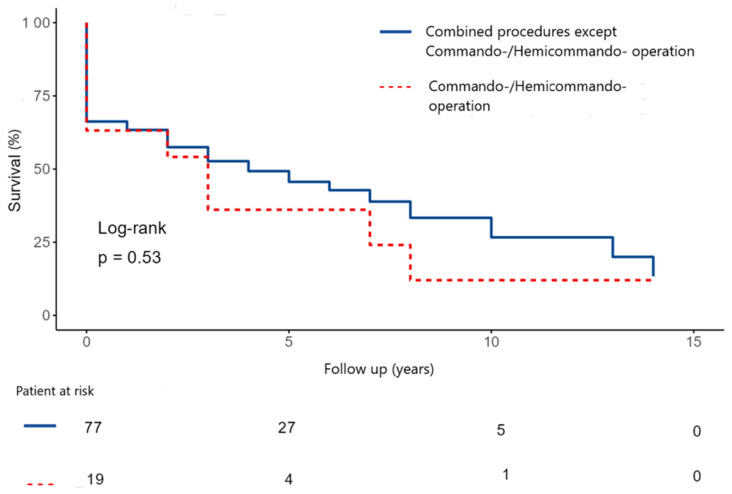
Kaplan–Meier curve shows survival after surgical treatment for extensive IE with abscess formation (except commando/hemi-commando) versus partial destruction of the heart skeleton (commando/hemi-commando). IE: Infective endocarditis.

The 5- and 10-year survival were 48.5 ± 6.0% and 30.5 ± 7.8% after commando/hemicommando operation vs. operations for extensive abscess, but without destroying the IVF (Figure 6). There was no statistically significant difference between the two groups.

**Figure 7:**
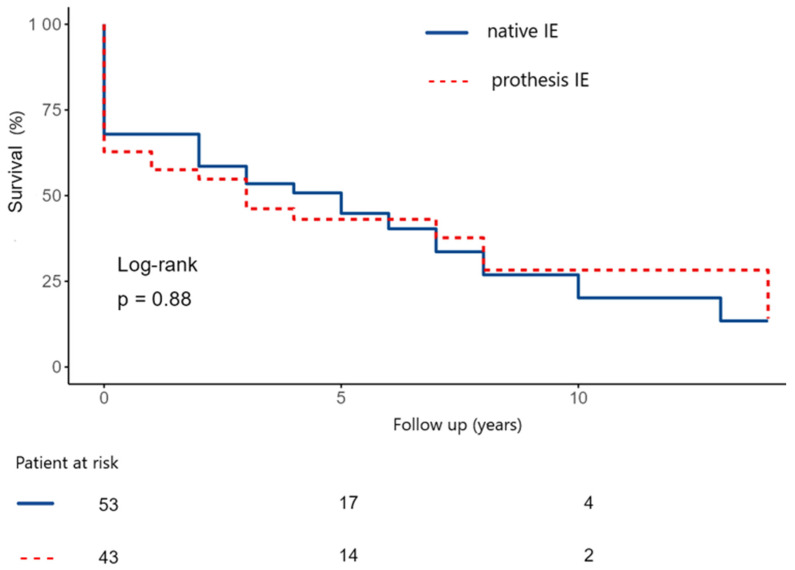
Kaplan–Meier curve depicting long-term survival in native and prothesis infective endocarditis. (IE: Infective endocarditis).

Furthermore, we compared the survival probabilities in relation to native and prosthesis IE (NVE vs. PVE). The estimated 5- and 10-year survival rate in the native IE group were 50 ± 7.5% and 24.5 ± 9.1% (Figure 7). In the prothesis IE group, the estimated 5- and 10-year survival rate were 41.8 ± 8.0 and 26.1 ± 10.2% (*p*-value: 0.88).

## 4. Discussion

To date, there are just several studies examining the results after the surgical treatment of IE [14,15,16]. Acute IE most commonly affects the aortic and mitral valves. Most interventions are isolated valve procedures [14]. However, there are few studies on extensive IE, especially when there is partial destruction of the cardiac skeleton.

This study investigated the early and long-term outcome after a complex surgical treatment of active IE, partially destroying the cardiac skeleton and necessitating patch reconstruction or involving at least two heart valves, over a 16-year period.

Gillinov et al. showed a significantly reduced mortality risk in the study, only including surgically treated patients with native endocarditis [15]. In Gillinov’s study, for example, there were no intrahospital deaths and 10-year survival was 73%. In our study, 24 patients died in the hospital and the 10-year survival was only 26.1%. This can be explained by the patient characteristic, which was significantly less ill in Gillinov’s study. In our study, 44% of patients had had previous cardiac surgery and 90% of patients were operated on urgently or emergently, which are both very important risk factors associated with poorer surgical outcomes [17,18]. Furthermore, only about half of the patients in the above-mentioned study had active endocarditis. Another crucial point is that in our study, 88% of patients presented with abscess formation, whereas in Gillinov’s study, only 11% of patients had abscess formation. This may explain the significant difference in mortality. Patients with active endocarditis tend to be sicker and generally have a significantly higher perioperative risk. This is also reflected in EuroSCORE II, which had a median of 12.7% (IQR 26.4) in our study population. Another decisive factor is that patients with abscess formation require a significantly more extensive and complex surgical procedure than other patients.

Regarding the pathogen spectrum, *Staphylococcus aureus* is the most common pathogen for IE in high-income countries, while oral *Streptococci* are becoming increasingly rare [1,19,20]. O’Connor was also able to prove this in his study [20]. In a study period from January 2005 to January 2014, O’Connor et al. examined 202 patients with infective endocarditis. *Staphylococci* were identified as the causative pathogen in 57%, of which *Staphylococcus aureus* was present in 36%, followed by *Streptococci* with 24.6% and *Enterococci* with 11%. This was also shown in our study, in which *Staphylococcus species* was detected in 49.5% and *Staphylococcus aureus* was detected in 34%, followed by *Steptococcus species* in 17.8% and *Enterococcus species* in 15.2%. In low-income countries, *Streptococci* and *Staphylococcus aureus* are predominant [21]. O’Connor also investigated pathogen detection in native valve endocarditis (NVE) and prosthetic valve endocarditis (PVE). In both groups, *Staphylococci* were far in the lead. *Streptococci* were detected significantly more frequently in the NVE group compared to the PVE group, whereas *Enterococci* was detected significantly more frequently in the PVE group. We were able to demonstrate similar results in our study. The proportion of *Streptococci* in the NVE group was 16.5%, compared with 3.8% in the PVE group. The detection of *Enterococci* in our study was similar in both groups, at 7.6%.

Navia et al. [22] examined 138 patients who underwent double valve replacement with the surgical reconstruction of IVF, in the sense of a commando or hemi-commando procedure, in a study period from 1988 to 2017. Of these, 83 patients underwent long-term follow-up. Recurrent IE was detected in 20% and reoperation had to be performed in 28% of cases. A total of 24% died after discharge. In our study, we were able to follow up 89 patients: recurrent IE was present in only 2.7% of our cohort and reoperation had to be performed in only 3.6%. Looking more closely at the cause of death, we were able to prove death due to cardiac causes in 24.3%. When comparing the two studies, however, it must be borne in mind that our study included only 19 patients who had undergone a commando/hemi-commando procedure.

Generally, looking at the cohort of commando and hemi-commando procedures, there are very few studies dedicated to this particularly sick patient population. Here, the study by Navia et al. is one of the largest studies. In Navia et al.’s study, the overall survival after 5 and 10 years was 48% and 37%, respectively. In our study, survival was comparable: 5-year survival was 48.5% and 10-year survival was 30.5%.

In terms of antibiotic treatment, aminoglycosides were long considered the antibiotic of choice, due to their strong bactericidal effect [23]. In the present study, aminoglycosides are also in second place in antibiotic treatment. However, the use of aminoglycosides has recently declined, not only because of their nephrotoxicity [23]. In addition, alternative antibiotic treatment strategies are now available. Oral antibiotic therapy is also becoming increasingly important. In our study, however, we found that only 76% of patients received any preoperative antibiotic therapy at all. In this respect, it can be concluded that the diagnosis of IE is still in need of improvement.

### Study Limitation and Strengths

This study belongs to one of few published studies with the data of patients, collected over a 16-year period, who underwent surgery for severe and destructive IE, requiring patch implantation.

This study has several limitations. The findings may contribute to establishing a consensus on the utility of PCR analysis in patients with infective endocarditis and may encourage a broader adoption of this diagnostic modality in clinical practice. However, this was a retrospective, single-center, non-randomized, observational study, and all inherent limitations of such a design apply. The sample size was relatively small. Additionally, the heterogeneity of surgical procedures limits direct comparisons between subgroups. Regarding statistical analyses, multivariate risk assessments should be interpreted with caution, due to the limited cohort size. The derived information could potentially help surgeons with decision-making. Future investigations should be conducted as multicenter studies to allow for the inclusion of a larger patient population. A prospective, and potentially randomized, study design would provide advantages over retrospective evaluations.

## 5. Conclusions

This study was able to demonstrate that in industrialized countries, extensive endocarditis with abscess formation or destruction of the heart skeleton is associated predominantly with *Staphylococcus aureus*. Patients who underwent a commando/hemi-commando procedure have no survival disadvantage compared to other patients, although the study may be underpowered to detect modest differences.

Similarly, there is no survival advantage with native valve compared to prosthetic valve IE when extensive abscess formation is present. However, the morbidity and mortality of these patients are still very high and they increase significantly with advanced findings, e.g., abscess formation. The patients are among the sickest in cardiac surgery and the therapy, both intra- and postoperative, requires special attention. Further studies, ideally multicentric and randomized, are warranted.

## Figures and Tables

**Table 1:** Demographics.

Variables	n = 96
Age (years), median (IQR)	68 (17)
**Gender**	
Female	29 (30%)
Male	67 (70%)
**Comorbidities**	
Coronary artery disease	38 (39%)
Diabetes	26 (27%)
COPD	12 (12%)
Atrial fibrillation	27 (28%)
Preoperative dialysis	11 (11%)
Stroke preoperatively	34 (35%)
**NYHA class** ≥ III	66 (69%)
**CCS** ≥ 3	8 (8%)
**LV EF** ≥ 30%	96 (99%)
**Kidney injury (KDIGO)** ≤ Stage G3 (GFR 30–60) [mL/min/1.73 m^2^]	51 (53%)
**EuroSCORE II (%)**, median (IQR)	12.7 (25.6)
**Aortic valve**	
Insufficiency (grade I–IV)	53 (55%)
Stenosis	16 (17%)
Combined	5 (5%)
**Mitral valve**	
Insufficiency (grade I–IV)	63 (65%)
Stenosis	8 (8%)
Combined	7 (7%)
**Tricuspid valve**	
Insufficiency (grade I–IV)	27 (28%)
Stenosis	0
**Pulmonary valve**	
Insufficiency (grade I–IV)	2 (2%)
Stenosis	0
**Urgency**	
Elective	10 (10%)
Urgent	63 (66%)
Emergency	23 (24%)

GFR: Glomerular filtration rate; LV EF: Left ventricular ejection fraction; and NYHA: New York Heart Association.

**Table 2:** Intraoperative details.

Variables	n = 96
Reoperation	43 (45%)
Abscess	85 (88%)
Aortic valve replacement	77 (80%)
Aortic valve repair	2 (2%)
Homograft in aortic position	2 (2%)
Bentall-de Bono operation	43 (45%)
Mitral valve replacement	30 (31%)
Mitral valve repair	21 (22%)
Tricuspid valve replacement	1 (1%)
Tricuspid valve repair	8 (8%)
Coronary artery bypass graft surgery	17 (18%)
Reconstruction of the aorto-mitral curtain	29 (30%)
Commando and hemi-commando operation	19 (20%)
Cardio-pulmonary bypass time (min), median (IQR)	176 (102)
Aortic cross-clamp time (min), median (IQR)	124 (74)

**Table 3:** Risk factors associated with higher and lower mortality.

	Hazard Ratio	95% CI	*p*-Value
COPD	2.276	4.958	0.038
Preoperative renal insufficiency	0.501	0.919	0.025
PAD	3.830	11.181	0.014
Coronary heart disease	1.978	3.386	0.013
Time of ICU stay	1.032	1.056	0.007
LVOT reconstruction	3.544	8.804	0.006
Ascending aortic replacement	2.053	3.972	0.033
Aortic valve repair	4.350	18.480	0.046
Mitral valve replacement	2.299	4.232	0.008
Tricuspid valve repair	3.255	8.725	0.019
Triple valve surgery	3.531	7.623	0.001
Postoperative dialysis	2.905	5.458	<0.001
Low cardiac output syndrome	3.100	5.878	<0.001
ECLS therapy	2.153	4.348	0.033
Multiple organ failure	3.413	7.940	0.004

All variables were included in a single regression model. Risk factor associated with higher mortality: hazard ratio (HR) < 1; risk factors associated with lower mortality: hazard ratio < 1. COPD: chronic obstructive pulmonary disease; PAD: peripheral arterial disease; ICU: intensive care unit; LVOT: left ventricular outflow tract; and ECLS: extracorporeal life support.

## Data Availability

Data supporting the reported results can be provided by the first author upon request.

## Abbreviations

The following abbreviations are used in this manuscript:

AML	Anterior mitral leaflet
AO	Aorta
AV	Aortic valve
CBP	Cardio-pulmonary bypass
CI	Confidence interval
COPD	Chronic obstructive pulmonary disease
CT	Computer tomography
ECLS	Extracorporeal life support
GFR	Glomerular filtration rate
HR	Hazard ratio
ICU	Intensive care unit
IE	Infective endocarditis
IQR	Interquartile range
IVF	Intervalvular fibrosa
LV EF	Left ventricular ejection fraction
LVOT	Left ventricular outflow tract
MV	Mitral valve
NVE	Native IE
NYHA	New York Heart Association
PAD	Peripheral arterial disease
PCR	Polymerase chain reaction
PVE	Prosthesis IE
